# The Biopsychosocial-Digital Approach to Health and Disease: Call for a Paradigm Expansion

**DOI:** 10.2196/jmir.9732

**Published:** 2018-05-18

**Authors:** Alireza Ahmadvand, Robert Gatchel, John Brownstein, Lisa Nissen

**Affiliations:** ^1^ School of Clinical Sciences Faculty of Health Queensland University of Technology Brisbane Australia; ^2^ Department of Psychology College of Science University of Texas at Arlington Arlington, TX United States; ^3^ Department of Pediatrics Harvard Medical School Boston, MA United States; ^4^ Computational Health Informatics Program Boston Children's Hospital Boston, MA United States

**Keywords:** digital health, digital technologies, Biopsychosocial Model to Health and Disease, human resources for health

## Abstract

Digital health is an advancing phenomenon in modern health care systems. Currently, numerous stakeholders in various countries are evaluating the potential benefits of digital health solutions at the individual, population, and/or organizational levels. Additionally, driving factors are being created from the customer-side of the health care systems to push health care providers, policymakers, or researchers to embrace digital health solutions. However, health care providers may differ in their approach to adopt these solutions. Health care providers are not assumed to be appropriately trained to address the requirements of integrating digital health solutions into daily everyday practices and procedures. To adapt to the changing demands of health care systems, it is necessary to expand relevant paradigms and to train human resources as required. In this article, a more comprehensive paradigm will be proposed, based on the ‘biopsychosocial model’ of assessing health and disease, originally introduced by George L Engel. The “biopsychosocial model” must be leveraged to include a “digital” component, thus suggesting a ‘biopsychosocial-digital’ approach to health and disease. Modifications to the “biopsychosocial” model and transition to the “biopsychosocial-digital” model are explained. Furthermore, the emerging implications of understanding health and disease are clarified pertaining to their relevance in training human resources for health care provision and research.

## Introduction

Digital health is an advancing phenomenon in modern societies, and their health care systems. The US Food and Drug Administration identifies “digital health” as, “a broad scope which includes mobile health (mHealth), health information technology, wearable devices, telehealth and telemedicine, and personalized medicine.” The US Food and Drug Administration perceives benefits of digital health to include expanded access to health care services, reduced health care system inefficiencies and costs, improved quality of care, enhanced self-management, and more personalized approaches towards medicine [1].

## Driving Factors of Digital Health and the “Digitally-Engaged Patient”

Currently, numerous stakeholders in developed and developing countries are continuously exploring and evaluating the potential benefits of digital health solutions at the individual, population, and/or organizational levels. The advancement of the digital health space has formed important driving factors including a progressive desire for innovation by industry players and developers of digital health solutions. Aspects of consideration is the increasing demand from societies to embrace more individualized, yet engaging, continuous health care services; the empowerment of health care system clients; and the growing diversity, affordability, and efficiency of digital health solutions.

Interestingly, these driving factors are creating “pull” effects from the customer-side, to ‘push’ health care providers, policymakers, or researchers to re-examine the traditional paradigms to ultimately address and embrace digital health solutions. Recent examples are the joint efforts by the US Department of Health and Human Services and the UK’s National Health Service for the adoption of digital health, and the adoption of principles that promote safe and effective mHealth applications by the American Medical Association [2,3]. Another implementation avenue of digital technologies has been in the management of chronic conditions such as pain, which has caused an influx of scientific literature on digital health solutions [4,5]. Additionally, the notion of ‘the digitally-engaged patient’ is emerging across health care policy, service provision, and research domains.

Digitally-engaged patients are demanding more independence in their choices, voices, and shared decision-making. They may demand more—sometimes radical—digital innovations in the delivery modes of health care services, evaluating diseases, psychological conditions and social behaviors, and analyzing generated health care data. However, health care providers may differ in their approaches to adopting digital health solutions. Specifically, health care providers’ knowledge, attitudes, skills, and practices regarding digital health may vary depending on the context or condition [6]. To maximize the benefits of modern digital technologies in improving patient outcomes, England has reported the need for clinicians to use their expertise to improve health care and redesigning care utilizing digital health [7]. Additionally, in the UK, studies have shown that investment in provision of additional training for both professionals and the public would help strengthen the normalization, uptake, and use of digital health and wellness services [8]. The World Health Organization’s mHealth Technical Evidence Review Group, provides another interesting example, by proposing the mHealth Evidence Reporting and Assessment checklist to boost both comprehensiveness and quality in the reporting by health care practitioners, on the effectiveness of digital health programs [9].

## Approaches to Digital Empowerment of Patients

To address the effectiveness, safety, and security of digital health solutions, it is still logical to expect that most of the information exchange and decision support at the individual level may essentially happen between health care providers and their patients during in-person or virtual visits. Nevertheless, no one can assume that health care providers are equipped or trained appropriately to responsibly react to the new demands for the usage of digital health solutions, and the requirements of their integration into usual daily practices. Due to these technological advances, it is necessary to re-evaluate and expand relevant paradigms used for training human resources for health care systems and service provision.

Scientific literature focused on the effects of digital empowerment paradigms on patients and their effects on health care provision highlights two strong mainstream approaches: “techno-utopian,” and “techno-critical.” “Techno-utopian” idealistically approaches the revolutionary effects of digital health with an optimistic perspective. Contrastingly, the “techno-critical” approach is more pragmatic, emphasizing the inherent complexities, both for patients and for health care providers, in managing health and disease conditions. The latter approach focuses on the possible associations between psychological and sociocultural dimensions of patients’ engagement in their own health care via digital health. The main dimensions may include: provoked emotions and possible resistance, regulatory and disciplinary issues, perceived contribution towards the burden of care, and the requirements for operation of “unseen work” on the part of health care workers, attributed to digital technology [10].

## Need for More Comprehensiveness About Digital Health

To suggest a more comprehensive approach, the biopsychosocial (BPS) model, introduced by George L Engel [11] has been adapted to include technology. This individual-level model of assessing health and disease, previously expanded in a heuristic manner by other scientists [12-14], must include a digital component. The proposed, “biopsychosocial-digital” model expands the traditional three-tier domains of understanding health and diseases (ie, biological, psychological, and social), in addition to training future human resources of health care systems. Furthermore, this model may act as a generic, neutral, and extended basis for the assessment of the possible effects and interactions of digital health technologies. Therefore, the hopes are that the “biopsychosocial-digital” model will bridge the techno-utopian and techno-critical approaches.

The BPS model emphasizes that the causes and outcomes of health and disease conditions should be considered in lieu with biological, psychological, and social factors. The model implies that for optimal management of conditions, the health care team should address all three influences on the patient. Subsequently, health care providers must be directly, or indirectly, familiar with applying this heuristic model for optimal care. Health care providers must acknowledge that the patients’ engagement in health and disease management is influenced by their concurrent medical conditions, psychological factors, and sociocultural barriers in the environment [11]. The BPS model has been widely used in different health-related disciplines to understand the nature of various disease conditions and the provision of training to professionals. Even though limitations exist with this model, most have been shown to be unfounded [15]. Limitations include the dichotomization between biology, psychology, and society, as well as the masking of an underlying biomedical approach [16]. Digital health technologies are expected to address these limitations by the provision of more accessible, scalable, and comprehensive data from various aspects of health and disease. Furthermore, the availability of various technologies which can continue to capture biological, psychological, and social information provide a non-dichotomized understanding of an individual’s medical condition and help in unmasking the biomedical focus of the original model.

## Reasons Behind the Call and Methodology for its Justification

In summary, to justify the BPS expansion, the fundamental effects of various digital health technologies on individual components of the original BPS model (ie, biological, psychological, and social) are discussed and supported by the provision of a number of pertinent examples. The potential mechanisms of how digital health may directly or indirectly affect the above-mentioned domains are beyond the scope of this current viewpoint manuscript. Nevertheless, one of the potential mechanisms involves the rapid changes in health care resulting from the adaptation of digital health technologies in various aspects of health and disease. In recent years, there have been increased efforts for the documentation, reflection, and analysis of the changes caused by digital health in the academic, critical psychosocial scientific literature [10].

A summary of the important reasons behind the paradigm expansion, from “biopsychosocial” to “biopsychosocial-digital” is provided below.

Firstly, individual-level digital biological data, both provider-generated and patient-generated, are becoming increasingly available and accessible to people and patients. As a fundamental example, DNA as a digital molecule is becoming the cornerstone of digital genomics services. Interestingly, portable biosensors have proven useful in providing information to support managing health and enabling affordable access to populations in low socioeconomic situations and/or remote geographical environments [17,18]. Additionally, the integration of personal self-tracking data with electronic medical records is already being implemented in some American hospitals [19]. Consequently, the digital “expansion” of one’s “biological self,” has become an expected outcome, as people are able to explore their biology through new methods.

Secondly, evidence shows that digital health solutions may directly or indirectly affect psychosocial processes that are part of the complex interactions of the individuals’ environments [20]. Digital health solutions may be additional sources of both positive and negative psychological reactions at the individual level (eg, personal reassurance from supported decisions by apps, or escalated anxiety induced by lost health data). Overall, digital health solutions have produced greater patient satisfaction [21]. Some authors have highlighted a paradigm shift, through digital technologies, in delivering psychological behavior change interventions [22]. As a result, health care providers should be systematically equipped to address these new psychological aspects of digital health solutions.

**Figure 1:**
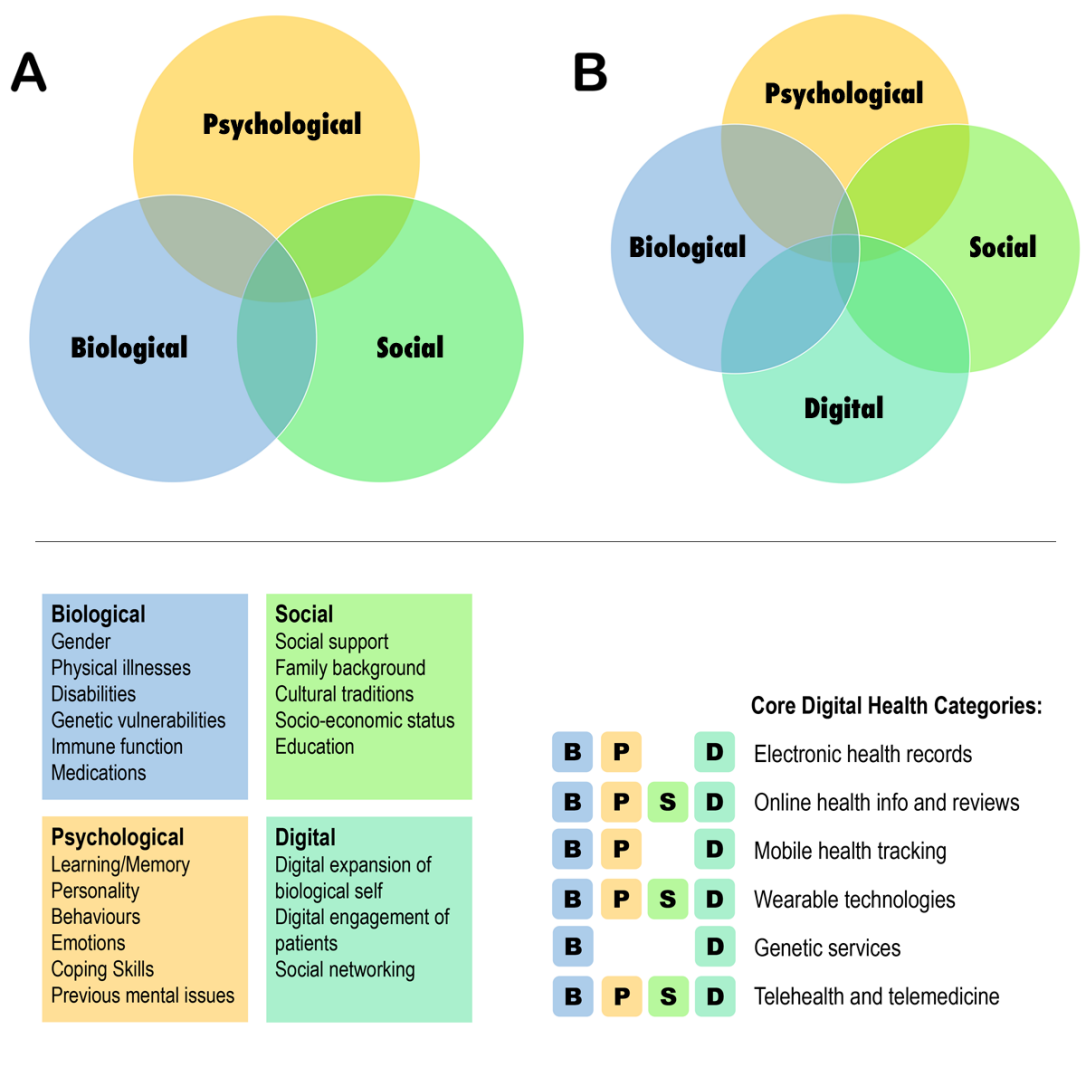
Comparison between the conventional biopsychosocial model (panel A) with our proposed paradigm (panel B), along with relevance of the proposed paradigm to six core digital health categories that operate within business to patient/consumer market contexts. B: biological; P: psychological; S: social; D: digital.

Thirdly, socialization around health, both at individual and group levels, has become increasingly popular in the digital space. The popularity of this trend shows both favorable and unfavorable effects as there is improved access to individuals in hard-to-reach sociocultural environments. Also, improving quality-of-care by patients’ social media inputs has been considered a promising area in US hospitals [23]. Conversely, social isolation because of breached privacy and hacked health data has become an increasing concern. Figure 1 compares the conventional biopsychosocial model (panel A) with the proposed biopsychosocial-digital model (panel B).

A three-step methodology provides justification of the biopsychosocial model expansion. The details of the methodology used are beyond the scope of this manuscript. In summary, a systematic review of literature was conducted to determine supporting evidence about the relationship between digital health technologies and independent or interrelated components of the biopsychosocial model. A gap analysis was performed to identify potential domains that could be incorporated to the original model. Lastly, an expert consensus report was conducted on the new model to provide feedback and critique through discussion.

## Implications of the Paradigm Expansion

The biopsychosocial-digital paradigm could have emerging implications in understanding health and disease and therefore, in training human resources for health care provision and research. Furthermore, the new biopsychosocial-digital paradigm offers a systematically-expanded means of addressing patients’ experiences of digital health solutions with their respective biological, psychological, and social aspects of health and disease.

Additionally, this expanded paradigm emphasizes why and how the knowledge of new digital health solutions, as they appear, is critical for training health care providers. Training is essential for improving health care providers’ understanding of the possible effects of digital health solutions on the three conventional aspects of health and disease. Subsequently, such effects can be successfully taught to health care providers [24]. Moreover, this paradigm goes beyond assessing acceptability, usability, and satisfaction of digital health solutions, allowing a deeper understanding of their usage. Insights from the biopsychosocial-digital paradigm helps anticipate and interpret the usage and outcomes of digital health solutions towards individualized health solutions. Interestingly, even legal frameworks such as Legal Challenges in Digital Health, support the development and evaluation of digital health services [25] and the proposed model further strengthens those frameworks.

The biopsychosocial-digital model further complements the growing trend of integrating digital health services through joined interdisciplinary teams, to provide better care and to address patients’ needs. Therefore, health care providers will consider the complex interactions among digital solutions and biological, psychological, and social aspects of health and disease, rather than the traditionally overly-simplistic biomedical-espoused causal and associated processes.

## Influencing Medical Education and Clinical Practice

The scientific literature is being enriched continuously with the possible implications of digital health technologies for medical education. Two important aspects are worth noticing: educating future health care practitioners about current and foreseeable technological innovations and enabling practitioners to adjust accordingly to future changes in their fields of specialty [26]. Specifically, in some disciplines, education is focused on the impact and advancements of modern digital technologies on patient care [27].

Various case studies discuss the incorporation of digital health technologies such as Blockchain use in health care, digital health device assessment, mobile health apps, pharmacogenomics and personalized medicine, use of electronic health records in practice, virtual reality and augmented reality, and wearable devices in clinical practice [26]. The importance of integrating digital technologies into medical education results in the improvement of interprofessional team dynamics, extended opportunities for inter-professional team education, and increased possibilities for interprofessional team practice. Moreover, health knowledge brokerage as another fundamental example of interprofessional collaboration and teaching health info-mediary activities from a patient-centered perspective are novel aspects of digital health incorporation into medical education [28]. The biopsychosocial-digital approach to health and disease benefits medical education initiatives by providing a more inclusive paradigm.

**Summary points from the biopsychosocial-digital model.**

- Digitally-engaged patients are demanding more independence, in their choices, voices, and shared decision-making.
- Health care providers’ knowledge, attitudes, skills, and practices regarding digital health may vary notably, depending on the context or condition.
- A newer biopsychosocial-digital paradigm offers a systematically-expanded means of addressing patients’ experiences of intended or unintended interactions of digital health solutions with their health and disease conditions.
- The new paradigm also helps in expanding the research and dialogue approaches to the perspectives of the digitally-engaged patients and to address the pros and cons of digital health solutions by health care providers.

The proposed paradigm has implications for the education of patients, as an important part of clinical practice. Recent efforts focused on the development of more comprehensive instruments to assess digital health literacy or eHealth literacy (eg, Digital Health Literacy Instrument) [29] will be better aligned with the biopsychosocial-digital model.

Certain methods might be used to support the integration of digital health concepts into medical education and clinical practice. Methods include systematic and regular revision of the educational curricula by medical universities, updating clinical practice guidelines and/or learning outcomes by professional medical colleges and providing examples, information, advice, and assistance about relevant digital health technologies. Moreover, the expansion of research efforts in different medical disciplines will increase the understanding of digital health utilization across the health and disease spectra whilst benefitting medical education and clinical practice. Textbox 1 summarizes key information from the biopsychosocial-digital model.

## Conclusions

### Limitations of the New Viewpoint

The original intention for proposing the new biopsychosocial-digital approach to health and disease was to generate further discussion and feedback. However, the ultimate expansion of the paradigm, requires future research beyond the scope of this manuscript to generate empirical data for potential substantiations of the proposed model. The new model provides value to future studies which explore the interrelationships between the different domains of the paradigm.

### Conclusion and Call for Action

The proposed biopsychosocial-digital paradigm helps expand the research and dialogue that accommodates the digitally-engaged perspective of patients around their health and disease. This model plausibly helps in the training of health care professionals by providing them a combination of clinical and digital skills. Professionals are able to benefit from the positive aspects of digital health solutions, address their challenges, and ultimately overcome negative aspects.

In the near future, an extension of research efforts to validate and provide feedback on this new biopsychosocial-digital paradigm, using data from real-world settings will be required. Furthermore, designing applied projects which address the integration of biopsychosocial-digital paradigm into the educational curricula across health and medical disciplines will be necessary for health care providers.

## Abbreviations

BPS: biopsychosocial
mHealth: mobile health
